# Practical Security of High-Dimensional Quantum Key Distribution with Intensity Modulator Extinction

**DOI:** 10.3390/e24040460

**Published:** 2022-03-26

**Authors:** Yang Wang, Ge-Hai Du, Yang-Bin Xu, Chun Zhou, Mu-Sheng Jiang, Hong-Wei Li, Wan-Su Bao

**Affiliations:** 1 National Laboratory of Solid State Microstructures, School of Physics and Collaborative Innovation Center of Advanced Microstructures, Nanjing University, Nanjing 210093, China; 2 Henan Key Laboratory of Quantum Information and Cryptography, SSF IEU, Zhengzhou 450001, China; dgh@qiclab.cn (G.-H.D.); xyb@qiclab.cn (Y.-B.X.); zc@qiclab.cn (C.Z.); jms@qiclab.cn (M.-S.J.); lhw@qiclab.cn (H.-W.L.); bws@qiclab.cn (W.-S.B.); 3 Synergetic Innovation Center of Quantum Information and Quantum Physics, University of Science and Technology of China, Hefei 230026, China

**Keywords:** quantum key distribution, high-dimensional, practical security, intensity modulator extinction

## Abstract

Quantum key distribution (QKD) has attracted much attention due to its unconditional security. High-dimensional quantum key distribution (HD-QKD) is a brand-new type of QKD protocol that has many excellent advantages. Nonetheless, practical imperfections in realistic devices that are not considered in the theoretical security proof may have an impact on the practical security of realistic HD-QKD systems. In this paper, we research the influence of a realistic intensity modulator on the practical security of HD-QKD systems with the decoy-state method and finite-key effects. We demonstrate that there is a certain impact in the secret key rate and the transmission distance when taking practical factors into security analysis.

## 1. Introduction

Quantum key distribution (QKD) [1,2] initiates a novel routine of secret key sharing between two distant parties (usually called Alice and Bob) in the presence of an eavesdropper (called Eve). Since the proposal of the first QKD protocol—BB84 protocol [1]—QKD has become the focus point of quantum information technology [3,4]. The unconditional security of QKD, which is guaranteed by the laws of quantum mechanics, has already been proved via different methods [5,6,7]. After the traditional BB84 protocol, various types of new QKD protocols have been proposed. Among these, high-dimensional quantum key distribution (HD-QKD) has garnered much attention due to its excellent capacity of encoding multiple bits on one single photon and strong tolerance to channel noise. In high-dimensional quantum key distribution systems, information is encoded on high dimensional degree of freedom of quantum state, such as time-energy entanglement [8,9,10], time-bin encoding [11,12], path [13,14] and orbital angular momentum [15,16,17]. Security proof for the HD-QKD protocol has also been established [18,19,20]. With the technological development of high-dimensional quantum state preparation and measurement, different HD-QKD schemes have achieved a number of record-breaking results in recent years [21,22,23]. Thereinto, time-bin based HD-QKD scheme [11,23] has realized a record high secret key rate and can offer security against general coherent attacks.

Unfortunately, practical devices in realistic QKD systems often present imperfections and rarely conform to theoretical security models [24,25]. Therefore, there is always a gap between the theory and practice of QKD. During the past decades, the practical security of QKD systems has been researched extensively. The eavesdropper can steal secret key information between Alice and Bob by seeking and utilizing side-channels introduced by different imperfect devices. For example, an imperfect phase modulator would introduce phase-remapping attacks [26], a realistic fiber beam splitter may provide convenience to wavelength attacks [27], and practical single photon detectors (SPDs) could be affected by time-shift attacks [28], faked state attacks [29] and detection blinding attacks [30,31]. Fortunately, efforts have been focused on proposing corresponding feasible countermeasures [32,33,34], and robust QKD protocols have been proposed, which are immune against detection-side-channel attacks, e.g., measurement-device-independent QKD (MDI-QKD) [35,36] and twin-field QKD (TF-QKD) [37,38,39,40]. On this account, it is of great significance to analyze how imperfections in realistic transmitters influence the practical security of QKD. Since the practical intensity modulators (IMs), which have been used in practical transmitters, are band limited, electrical signal distortion may cause intensity fluctuations of pulses and other phenomena [41,42]. Therefore, it is important to quantitatively evaluate the imperfections in IMs for the security certification of practical QKD systems.

Analogously, there is also a deviation between theoretical security and practical performance in HD-QKD systems. Although theoretical security analysis for HD-QKD protocol is exhaustive, its practical feasibility is far from sufficient. Toward this end, research on the practical security analysis of HD-QKD protocol is ongoing. In realistic experimental implementations, the requirement for single photon sources is not easily satisfied, the weak coherent source is employed instead. This kind of source contains multi-photon signals and the eavesdropper can carry out the photon number-splitting (PNS) attack [43,44] to steal secret keys. Zhang et al. [45] applied decoy-state methods [46,47,48] to the HD-QKD protocol to defeat the PNS attack with an infinite number of decoy states and proved its security against collective attacks. Afterwards, the security analysis of the HD-QKD protocol employing a practical number of decoy states is followed [49]. In addition, the number of transmitted signals is always finite in practical QKD processes. This would bring in another practical issue: finite-key problem. There exists fluctuations between practical measurement output and theoretical estimation and the secret key rate would be calculated by mistake. Scarani et al. [50] and Tomamichel et al. [51] analyzed the practical finite-key security of the BB84 protocol under collective attacks and coherent attacks at the first step. Following the methods proposed in refs. [50,51], the practical security of decoy-state HD-QKD protocol against collective attacks [52] and coherent attacks [53] in the finite-key scenario is established. In decoy-state methods, the intensities of signal state and decoy states should be stable and controllable. Nonetheless, an unstable source would lead to intensity fluctuations in practical QKD systems. When there exist intensity fluctuations, the original characterization of the decoy-state method needs further improvement [54,55]. The effects of both intensity fluctuations of sources and statistical fluctuations have been discussed [56], and the results on the secret key rate were then further improved [57]. Following the approach to describing the intensity fluctuations proposed by Wang et al. [55,56], tight finite-key analysis for practical decoy-state QKD protocols with unstable sources is proposed [58,59,60]. To guarantee its practical performance, the practical security analysis of HD-QKD protocol needs further investigations.

In realistic implementations, the intensity modulator on Alice’s side is used to attenuate the light intensity and filter out some redundant light pulses. One important parameter of IM is the extinction ratio, which appears as a fixed finite value (ranging from 20 dB to 40 dB usually). In ref. [61], the finite extinction of imperfect intensity modulators in the BB84 system was investigated. It is surprising that the extra noise caused by realistic IM reduces Eve’s information. The secret key rate is increased and practical security is enhanced. To investigate the practical security of the HD-QKD protocol, we focus our attention on the impact of this realistic imperfection on practical HD-QKD systems in this work. We characterize a model of extinction ratio and derive a new expression of quantum bit error rates for HD-QKD. Then, the maximal tolerable quantum bit error rate and secret key rate are calculated for HD-QKD with the single photon state and the decoy-state method, respectively. The combined effect of the finite extinctions of intensity modulator and intensity fluctuations of the source in the finite-key scenario is analyzed as well.

The rest of this paper is organized as follows. In Section 2, we present a brief introduction on the state preparation and transmission processes of the HD-QKD system and establish the model characterization of the extinction ratio. In Section 3, we analyze the practical security of HD-QKD system with the single-photon source and the decoy-state method, respectively. Some further discussions on the combined influence of the finite extinction and intensity fluctuations on the practical security of HD-QKD system were also put forward. Simulation results are depicted in Section 4 and some conclusive comments are summarized in Section 5.

## 2. Model Characterization of the Extinction Ratio

**Figure 1:**
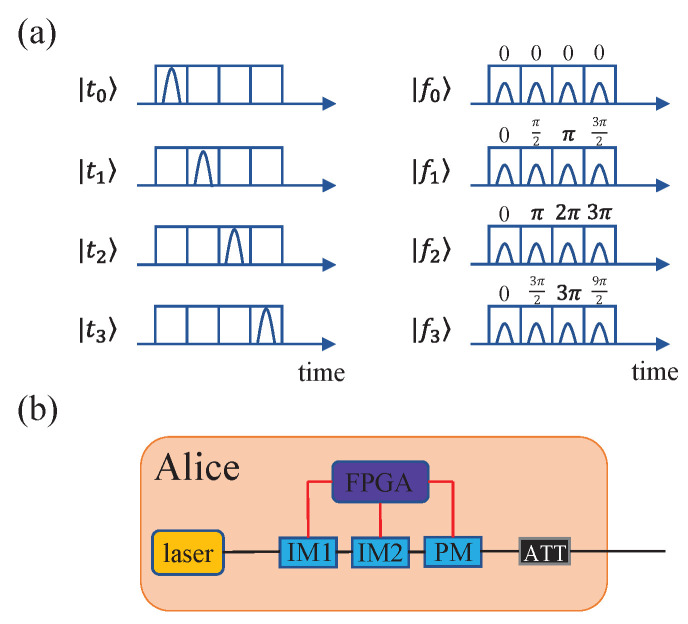
(Color online) Schematic diagram of the four-dimensional time-bin HD-QKD system. (**a**) Representation of time-bin states (left) and phase states (right). (**b**) Diagram of producing time-bin states and phase states on Alice’s side, where laser means Alice produces periodic light pluses with a laser source, FPGA is the field-programmable gate array, IM1 and IM2 are intensity modulators, PM is the phase modulator, and ATT is the attenuator. See more details in ref. [11].

Without a loss of generality, we take the four-dimensional time-bin HD-QKD scheme [11] for example. As illustrated in Figure 1, Alice employs two intensity modulators and one phase modulator controlled by a field programmable gate array (FPGA) to fabricate time-bin states $|t_{n}\rangle$ and phase states $|f_{n}\rangle$, which are the discrete Fourier transforms of time-bin states where $|f_{n}\rangle =\frac{1}{2}\sum_{m=0}^{3}exp\left(\frac{\pi inm}{2}\right)|t_{m}\rangle ,n=0,1,2,3$. Alice modulates a periodic chain of optical pulses produced by the laser source with IM1 to determine these pulses for either time-bin states or phase states. Each state consists of four time-bins and each time-bin contains one light pluse. For time-bin states, three light pulses out of four that we do not need are filtered out with IM1. Afterwards, IM2 is used to adjust the amplitude of phase states relative to the primary time-bin states and the phase modulator is used to encode different phase states. An attenuator is used to reduce the photon states to single-photon levels. In a realistic setup, the extinction ratio of the intensity modulator is not infinite; hence, light pulses cannot be attenuated to zero intensity and are filtered out thoroughly. Because phase states are not required to be attenuated to zero intensity, only the finite extinction of IM1 will have impact on the final security of the system.

Due to the electro-optic effect, we can apply different voltages on the intensity modulator to control the intensity of the passing light. Then, we can define the state of IM1 as “off” when it has high attenuation and “on” when it has nearly no attenuation. The light intensity is attenuated by a factor of $\sqrt{P_{off}}$ or $\sqrt{P_{on}}$ when the state of IM1 is off or on, and the intensity of the output light is $I_{off}=P_{off}I_{0}$ or $I_{on}=P_{on}I_{0}$, respectively, where $I_{0}$ is the intensity of the input light. Therefore, we can define the extinction ratio of the intensity modulator as follows.

(1) $$r=\frac{I_{on}}{I_{off}}.$$

Supposing that Alice prepares the signal state $|t_{0}\rangle$, high attenuation will be applied to the second, third and fourth time-bins. Let $a=\sqrt{P_{on}}$ and $b=\sqrt{P_{off}}$, the signal state transmitted out of IM1 can be written as follows:

(2) $$\begin{array}{cc} \; & \rho_{0}=a^{2}|t_{0}\rangle \langle t_{0}|+b^{2}(|t_{1}\rangle \langle t_{1}\left|+\right|t_{2}\rangle \langle t_{2}\left|+\right|t_{3}\rangle \langle t_{3}|)\\ \; & =\left(a^{2}-b^{2}\right)|t_{0}\rangle \langle t_{0}|+4b^{2}\cdot \frac{|t_{0}\rangle \langle t_{0}\left|+\right|t_{1}\rangle \langle t_{1}\left|+\right|t_{2}\rangle \langle t_{2}\left|+\right|t_{3}\rangle \langle t_{3}|}{4}\\ \; & =\left(a^{2}+3b^{2}\right)(\frac{a^{2}-b^{2}}{a^{2}+3b^{2}}|t_{0}\rangle \langle t_{0}|+\frac{4b^{2}}{a^{2}+3b^{2}}\cdot \frac{\hat{I}}{4})\\ \; & =\left(a^{2}+3b^{2}\right)\left(\frac{a^{2}-b^{2}}{a^{2}+3b^{2}}\rho_{ideal}+\frac{4b^{2}}{a^{2}+3b^{2}}\rho_{noise}\right) \end{array}$$

where $\rho_{ideal}$ denotes the pure signal state ($|t_{0}\rangle \langle t_{0}|$, $|t_{1}\rangle \langle t_{1}|$, $|t_{2}\rangle \langle t_{2}|$ or $|t_{3}\rangle \langle t_{3}|$), and $\rho_{noise}=\frac{\hat{I}}{4}$ denotes the density matrix of the extra noise introduced by the finite extinction ratio of IM1. After normalization, Equation (2) can be generally written as follows:

(3) $$\rho_{signal}=\frac{r-1}{r+3}\rho_{ideal}+\frac{4}{r+3}\rho_{noise}$$

since $r=\frac{I_{on}}{I_{off}}=\frac{P_{on}}{P_{off}}=\frac{a^{2}}{b^{2}}$. Similarly, we can extend the discussion above to arbitrary *d*-dimensional time-bin QKD systems. By calculation, the equation of state transmission can be written as follows.

(4) $$\rho_{signal}=\frac{r-1}{r+d-1}\rho_{ideal}+\frac{d}{r+d-1}\rho_{noise}.$$

By using this method, we can notice that (3) is of the same form as Equation (3) in ref. [61]. This is because, in the polarization coding BB84 protocol, *X* and *Z* basis states are generated and detected in the same manner and can transform mutually into each other. All four states are affected by the finite extinction of intensity modulators. On the other hand, in the time-bin HD-QKD protocol, time-bin states $|t_{n}\rangle$ and phase states $|f_{n}\rangle$ are neither generated nor detected in the same manner [11]. One basis cannot transform into the other mutually either. More importantly, only four time-bin states are affected by the finite extinction of intensity modulator in the state preparation process.

## 3. Security Analysis

In this section, we analyze the practical security of the time-bin HD-QKD system described above. We firstly analyze the practical security in the case of the HD-QKD system with the ideal single photon source. Then, we generalize our analysis to the HD-QKD system combined with the decoy-state method. The combined effect of intensity fluctuations in the laser source and the finite extinction of the intensity modulator is discussed in the end.

### 3.1. HD-QKD with the Single Photon State

Here, we discuss the universal situation for arbitrary *d*-dimensional HD-QKD protocol. The secret key rate of *d*-dimensional QKD system in asymptotic infinite-key scenario can be written as follows:

(5) $$R_{\infty }=\log_{2}d-H\left(e\right)-H\left(e_{p}\right)$$

where *e* is the quantum bit error rate (QBER) caused by noises and $H\left(x\right)=-x\log_{2}\left(\frac{x}{d-1}\right)-\left(1-x\right)\log_{2}\left(1-x\right)$ is the *d*-dimensional Shannon entropy [19]. After key sifting processes, Alice and Bob should perform classical post-processing, which consists of error correction and privacy amplification. The fractions H(e) of the sifted key bits are sacrificed to perform error correction, and the fractions $H(e_{p})$ of the sifted key bits are sacrificed to perform privacy amplification [62].

Since the finite extinction of IM1 will bring in some extra noises, QBER can be modified into the following.

(6) $$\begin{array}{cc} \; & e^{{}^{\prime }}=\frac{r-1}{r+d-1}e+\frac{d}{r+d-1}\cdot \frac{d-1}{d}\\ \; & =\left(1-\frac{d}{r+d-1}\right)e+\frac{d-1}{r+d-1}. \end{array}$$

The first term of the right hand side of (6) is caused by the noises introduced by Eve when she attempts to steal secret key information on the quantum channel via some attacking strategies. In addition, the second term of the right hand side is attributed to a probability of $\frac{d-1}{d}$ of an incorrect alphabet resulting from $\rho_{noise}$. Since the identity matrix remains unchanged under all unitary operations, Eve cannot achieve any useful information by performing any operation on $\rho_{noise}$. Therefore, the privacy amplification process is not required for the part of $\rho_{noise}$, and only a fraction $\left(1-\frac{d}{r+d-1}\right)$ of sifted key bits need to perform privacy amplification [62]. Certainly, all error bits should undergo error correction process and the the QBER is $e^{{}^{\prime }}$ now. Therefore, (5) turns into the following.

(7) $$\begin{array}{cc} \; & R_{\infty }^{{}^{\prime }}=\log_{2}d-H\left(e^{{}^{\prime }}\right)-\left(1-\frac{d}{r+d-1}\right)H\left(e\right)\\ \; & =\log_{2}d-H\left(e^{{}^{\prime }}\right)-\left(1-\frac{d}{r+d-1}\right)H\left(\frac{e^{{}^{\prime }}-\frac{d-1}{r+d-1}}{1-\frac{d}{r+d-1}}\right). \end{array}$$

### 3.2. HD-QKD with the Decoy-State Method

In this subsection, we only consider four-dimensional time-bin QKD systems with finite-key analysis. Islam et al. [11] applied decoy state methods to a four-dimensional time-bin QKD scheme and bounded the secret key length (denoted by *l*) as follows:

(8) $$l\leq max\lfloor 2{\tilde{s}}_{T,0}+{\tilde{s}}_{T,1}\left[c-H\left(\lambda^{U}\right)\right]-leak_{EC}+\Delta_{FK}\rfloor$$

where ${\tilde{s}}_{T,0}$ and ${\tilde{s}}_{T,1}$ are the vacuum and single-photon detection counts in the temporal basis, respectively. *c* is defined as $c:=-\log_{2}max_{i,j}|\langle f_{i}|t_{j}{\rangle |}^{2}$, and $\lambda^{U}$ is an upper bound of the single-photon phase error rate. $leak_{EC}=1.16H\left(x\right)$ is the number of secret key bits sacrificed for error correction processes, and $\Delta_{FK}$ is the finite-key estimation item. The detailed expressions of ${\tilde{s}}_{T,0}$, ${\tilde{s}}_{T,1}$ and $\lambda^{U}$ are given by the following:

(9) $$\begin{array}{cc} \; & {\tilde{s}}_{T,0}:=max\left\{\left\lfloor \frac{\tau_{0}}{\mu_{2}-\mu_{3}}\left(\frac{\mu_{2}e^{\mu_{3}}n_{T,\mu_{3}}^{-}}{p_{\mu_{3}}}-\frac{\mu_{3}e^{\mu_{2}}n_{T,\mu_{2}}^{+}}{p_{\mu_{2}}}\right)\right\rfloor ,0\right\},\\ \; & {\tilde{s}}_{T,1}:=max\{\frac{\mu_{1}\tau_{1}}{\mu_{1}\left(\mu_{2}-\mu_{3}\right)-\left(\mu_{2}^{2}-\mu_{3}^{2}\right)}[\frac{e^{\mu_{2}}n_{T,\mu_{2}}^{-}}{p_{\mu_{2}}}\\ \; & -\frac{e^{\mu_{3}}n_{T,\mu_{3}}^{+}}{p_{\mu_{3}}}+\frac{\mu_{2}^{2}-\mu_{3}^{2}}{\mu_{1}^{2}}\left(\frac{{\tilde{s}}_{T,0}}{\tau_{0}}-\frac{e^{\mu_{1}}n_{T,\mu_{1}}^{+}}{p_{\mu_{1}}}\right)],0\},\\ \; & \lambda^{U}:=\frac{{\tilde{v}}_{F,1}}{{\tilde{s}}_{F,1}}+\sqrt{\frac{\left({\tilde{s}}_{T,1}+{\tilde{s}}_{F,1}\right)\left({\tilde{s}}_{F,1}+1\right)}{{\tilde{s}}_{T,1}{\left({\tilde{s}}_{F,1}\right)}^{2}}ln\frac{2}{\beta }} \end{array}$$

where ${\tilde{v}}_{F,1}=\frac{\tau_{1}}{\mu_{2}-\mu_{3}}\left(\frac{e^{\mu_{2}}m_{F,\mu_{2}}^{+}}{p_{\mu_{2}}}-\frac{e^{\mu_{3}}m_{F,\mu_{3}}^{-}}{p_{\mu_{3}}}\right)$ and $\tau_{n}=\sum_{k\in K}e^{-k}\frac{k^{n}p_{k}}{n!}$, while ${\tilde{s}}_{F,1}$ has a similar form to ${\tilde{s}}_{T,1}$. One signal state and two decoy states are denoted as $K:=\{\mu_{1},\mu_{2},\mu_{3}\}$ (chosen with probabilities $p_{\mu_{1}}$, $p_{\mu_{2}}$ and $p_{\mu_{3}}:=1-p_{\mu_{1}}-p_{\mu_{2}}$, respectively) where $\mu_{1}>\mu_{2}+\mu_{3}$ and $\mu_{1}\geq \mu_{2}\geq \mu_{3}\geq 0$. More details are depicted in ref. [11]. In the finite-key scenario, there are statistical fluctuations in the parameter estimation procedure. Therefore, we employ the improved Chernoff bound to estimate the upper and lower bounds for $n_{T,k}$, which represents the total number of detection events in the temporal basis. For the improved Chernoff bound [63], the upper and lower bounds for the measurement outcome $n_{T,k}$ can be expressed as follows:

(10) $$\begin{array}{cc} \; & n_{T,k}⩽\frac{n_{T,k}}{1-\delta_{C}^{U}\left(n_{T,k},\varepsilon_{C}\right)}=n_{T,k}^{+}\\ \; & n_{T,k}⩾\frac{n_{T,k}}{1+\delta_{C}^{L}\left(n_{T,k},\varepsilon_{C}\right)}=n_{T,k}^{-} \end{array}$$

with probability of at least $1-2\varepsilon_{C}$, where $\varepsilon_{C}$ is the correctness parameter. For $\delta_{C}^{U}\left(x,y\right)$ and $\delta_{C}^{L}\left(x,y\right)$, a simplified analytical approximation is given by [63] the following.

(11) $$\begin{array}{c} \delta_{C}^{U}\left(x,y\right)=\delta_{C}^{L}\left(x,y\right)=\frac{-3ln\left(\frac{y}{2}\right)+\sqrt{{\left[ln\left(\frac{y}{2}\right)\right]}^{2}-8xln\left(\frac{y}{2}\right)}}{2x+2ln(\frac{y}{2})}. \end{array}$$

Substituting (10) and (11) into (9), we can derive a lower bound on the secret key’s length.

Because only the imperfections of IM1 have an effect on the practical security of this system and IM1 only acts on time-bin states, only error events in the temporal basis in (8) should append the consideration of the finite extinction of the intensity modulator. As discussed above, $leak_{EC}=1.16H\left(x\right)=1.16H\left(E_{t}\right)$, where $E_{t}=\frac{m_{T,\mu_{1}}+m_{T,\mu_{2}}+m_{T,\mu_{3}}}{n_{T,\mu_{1}}+n_{T,\mu_{2}}+n_{T,\mu_{3}}}$. In this fraction, $m_{T,k}=p_{\mu_{k}}p_{T}^{2}N\left(e_{d}\left(1-e^{-\eta \mu_{k}}\right)+0.75P_{d}\right)$ ($K\in \{\mu_{1},\mu_{2},\mu_{3}\}$) represents error events in the temporal basis, while $n_{T,k}$ is defined as $n_{T,k}=p_{\mu_{k}}p_{T}^{2}N\left(1-e^{-\eta \mu_{k}}+P_{d}\right)$. Here, $p_{T}$ is the preparation probability of time-bin states, η is the overall system transmittance and $P_{d}$ denotes the dark count rate of single photon detectors. $e_{d}$ is the error bit rate caused by the misalignment of the system, which includes the error bit rate introduced by the imperfect IM and can be obtained from the field test experiments. With another form of (6), i.e., $e=\frac{e^{{}^{\prime }}-\frac{d-1}{r+d-1}}{1-\frac{d}{r+d-1}}$, we can set d=4 and transform $e_{d}$ into the following.

(12) $$e_{d}\to \frac{e_{d}^{{}^{\prime }}-\frac{3}{r+3}}{1-\frac{4}{r+3}}.$$

Substituting (12) into (8), we can obtain a modified formula of secret key length while considering the finite extinction of the practical intensity modulator. Taking the number of transmitted signals *N* and state preparation rate into account, we can obtain the final secret key rate.

### 3.3. HD-QKD with Both Intensity Fluctuations and the Finite Extinction

Last but not least, we should mention that we carry out our security analysis with the assumption that there is no intensity fluctuations in the light exiting from the laser source. In reality, intensity fluctuations in practical QKD systems always exist and have deep influence on the performance of the HD-QKD system [58,59]. Taking both intensity fluctuations resulted from the unstable source and finite extinction of intensity modulator into consideration in the security analysis, we can figure out how these two issues affect the practical performance of the HD-QKD system simultaneously in a realistic setup. When conducting security analysis, we should employ Azuma’s inequality [64] instead of the improved Chernoff bound to estimate the statistical fluctuations caused by intensity fluctuations. This is because the intensity fluctuations would break the independent condition for independent random samples and Azuma’s inequality can hold with dependent random samples by the square. In greater detail, we use Azuma’s inequality to quantify the fluctuation ranges in ${\tilde{s}}_{T,0}$, ${\tilde{s}}_{T,1}$ and $\lambda^{U}$ in (9). With Azuma’s inequality, the observed values of the number of detection events and the observed number of errors for the case with intensity fluctuations in the temporal basis $n_{T,k}^{*}$ and $m_{T,k}^{*}$ satisfy the following:

(13) $$|n_{T,k}^{*}-n_{T,k}|\leq \delta \left(n_{T},\beta \right)$$

and

(14) $$|m_{T,k}^{*}-m_{T,k}|\leq \delta \left(m_{T},\beta \right)$$

with probability of at least 1−2β where $\delta \left(x,y\right)=\sqrt{2xln(\frac{1}{y})}$. Then, we find the following:

(15) $$\begin{array}{cc} \; & n_{T,k}^{*}\leq n_{T,k}+\delta \left(n_{T},\beta \right)=n_{T,k}^{+},\\ \; & n_{T,k}^{*}\geq n_{T,k}-\delta \left(n_{T},\beta \right)=n_{T,k}^{-},\\ \; & m_{T,k}^{*}\leq m_{T,k}+\delta \left(m_{T},\beta \right)=m_{T,k}^{+},\\ \; & m_{T,k}^{*}\geq m_{T,k}-\delta \left(m_{T},\beta \right)=m_{T,k}^{-} \end{array}$$

and they are the upper and lower bounds of $n_{T,k}^{*}$ and $m_{T,k}^{*}$ for all values of *k*, which appear in (9). Values for phase basis $n_{F,k}^{*}$ and $m_{F,k}^{*}$ hold a similar form to (13)–(15).

According to refs. [58,59], we assume that the fluctuation ranges of intensity $k\in (k^{-},k^{+})$ is known to Alice and Bob. We can present a detailed decoy-state analysis with intensity fluctuations. For $k\in \mu_{1},\mu_{2},\mu_{3}$, we also assume that $\mu_{1}>\mu_{2}+\mu_{3}$ and $\mu_{1}\geq \mu_{2}\geq \mu_{3}\geq 0$. When considering intensity fluctuations, we can obtain the lower bound for the number of vacuum events, and it is given by the following:

(16) $$\begin{array}{c} {\tilde{s}}_{T,0}:=max\left\{\left\lfloor \frac{\tau_{0}}{\mu_{2}^{-}-\mu_{3}^{+}}\left(\frac{\mu_{2}^{-}e^{\mu_{3}^{+}}n_{T,\mu_{3}}^{-}}{p_{\mu_{3}}}-\frac{\mu_{3}^{+}e^{\mu_{2}^{-}}n_{T,\mu_{2}}^{+}}{p_{\mu_{2}}}\right)\right\rfloor ,0\right\}, \end{array}$$

and the lower bound for the number of single-photon events can be expressed as follows.

(17) $$\begin{array}{cc} \; & {\tilde{s}}_{T,1}:=max\{\frac{\mu_{1}^{-}\tau_{1}}{\mu_{1}^{-}\left(\mu_{2}^{+}-\mu_{3}^{-}\right)-\left({\left(\mu_{2}^{+}\right)}^{2}-{\left(\mu_{3}^{-}\right)}^{2}\right)}[\frac{e^{\mu_{2}^{+}}n_{T,\mu_{2}}^{-}}{p_{\mu_{2}}}\\ \; & -\frac{e^{\mu_{3}^{-}}n_{T,\mu_{3}}^{+}}{p_{\mu_{3}}}+\frac{{\left(\mu_{2}^{+}\right)}^{2}-{\left(\mu_{3}^{-}\right)}^{2}}{{\left(\mu_{1}^{-}\right)}^{2}}\left(\frac{{\tilde{s}}_{T,0}}{\tau_{0}}-\frac{e^{\mu_{1}^{-}}n_{T,\mu_{1}}^{+}}{p_{\mu_{1}}}\right)],0\}. \end{array}$$

Substituting (9) and (15)–(17) into (8), we can derive the modified secret key rate formula by considering intensity fluctuations and the finite extinction of the intensity modulator simultaneously.

## 4. Simulation Results

**Figure 2:**
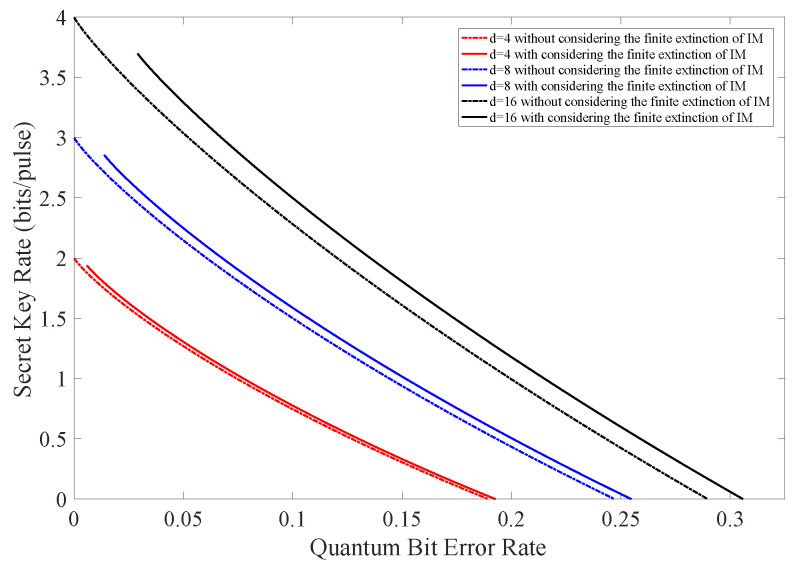
(Color online) The secret key rate vs. observed quantum bit error rate with and without considering the finite extinction of intensity modulator.

Figure 2 is the numerical simulation result of (7) where $e^{{}^{\prime }}$ is the observed quantum bit error rate. The extinction ratio *r* is selected as 500 (27 dB), which is a typical parameter value of practical devices.

As illustrated in Figure 2 and Table 1, we find that there is an increase in the maximal tolerable QBER when taking the finite extinction of the intensity modulator into account. Furthermore, this increase is more obvious for higher dimension *d*. This is because systematic noises come from two parts: Eve’s attacking behaviour and the imperfection of the practical intensity modulator, as illustrated in (6). When performing the error correction, all errors are considered to be introduced by Eve and Eve will lose more information than Alice and Bob. As a consequence, Alice and Bob can distill more secret keys, and the secret key rate is increased. We should also notice that three full curves in Figure 2 do not start from zero on the horizontal axis. This is because there exist intrinsic noises of $\frac{d-1}{r+d-1}$ caused by the imperfection of the practical intensity modulator when setting e=0 in (6).

**Figure 3:**
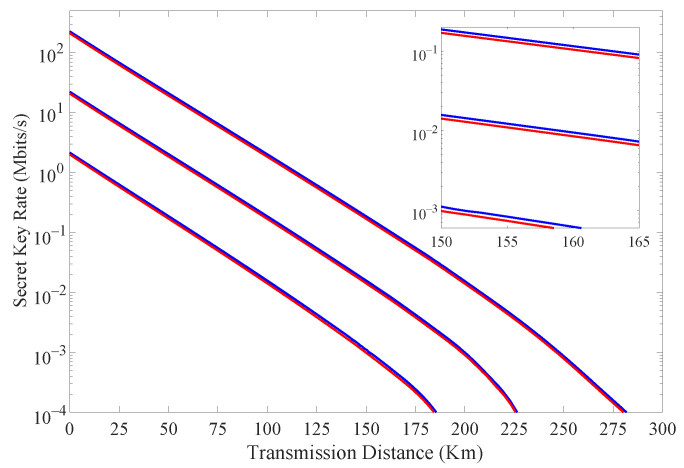
(Color online) The final secret key rate vs. transmission distance with (blue curves) and without (red curves) considering the finite extinction of intensity modulator for $N=6.25\times 10^{x}$ with *x* = 9, 10, 11 (curves from bottom to top).

Figure 3 shows the simulation results of the secret key rate with and without considering the finite extinction of the intensity modulator when Alice transmits different numbers of signals *N*’s. The simulation parameters are selected as follows. The average intensities of one signal state and two decoy states are selected to be 0.66, 0.16 and 0.002, respectively. The probabilities of sending these three states are 0.8, 0.1 and 0.1, respectively. Time-bin and phase states are prepared with probabilities of 0.90 and 0.10, which are $p_{T}$ and $p_{F}$. The quantum channel is described by a loss $\eta_{ch}=10^{-\alpha L/10}$, where α=0.2 dB/km is the loss coefficient of the fiber, and *L* (km) is the transmission distance. We also assume that the dark count rate $P_{d}=10^{-8}$ and two correctness parameters $\beta =1.72\times 10^{-10}$, $\varepsilon_{C}=10^{-12}$ from ref. [11]. As shown in Figure 3, we can see that when considering the finite extinction of the intensity modulator, the transmission distance increases about 1 km for different *N*’s. For $N=6.25\times 10^{11}$, there is an increase of 9–11% in the secret key rate, as illustrated in Table 2.

**Figure 4:**
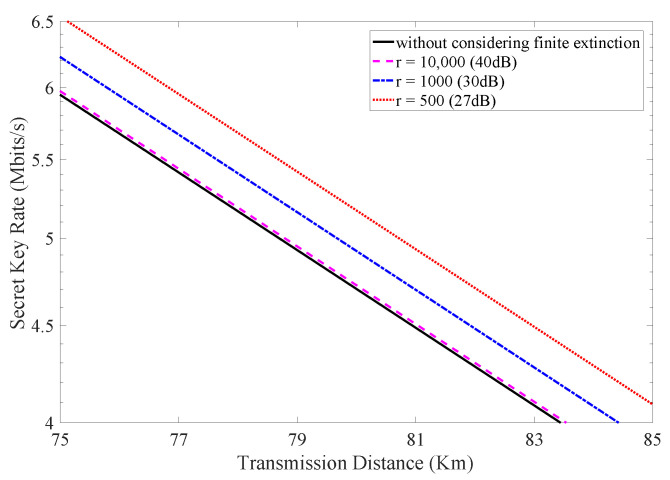
(Color online) The secret key rate vs. transmission distance considering different extinction ratios.

Furthermore, the influence of different extinction ratios on the practical performance of the HD-QKD system is investigated. We again employ the parameters of four-dimensional time-bin QKD system mentioned above and the number of transmitted signals is set to be $N=6.25\times 10^{11}$. The secret key rate results when considering different extinction ratios are depicted in Figure 4. It is beyond expectation that the lower extinction ratio can result in a higher secret key rate. This is because that the quantum bit error rate results from two parts: the imperfection of the practical intensity modulator and the channel noises. Different extinction ratios will make error rates resulting from these two factors make up different accounts for the total quantum bit error rate. For the HD-QKD system with lower extinction ratios, the intrinsic noises caused by the imperfection of practical intensity modulator appear higher. On the basis of the discussion above, Eve would lose more information during classical data post-processing. Therefore, the secret key rate becomes higher as a result. It should be noted that this conclusion can only be drawn when the total quantum bit error rate remains unchanged. HD-QKD systems with different quantum bit error rate values cannot be compared with each other.

**Figure 5:**
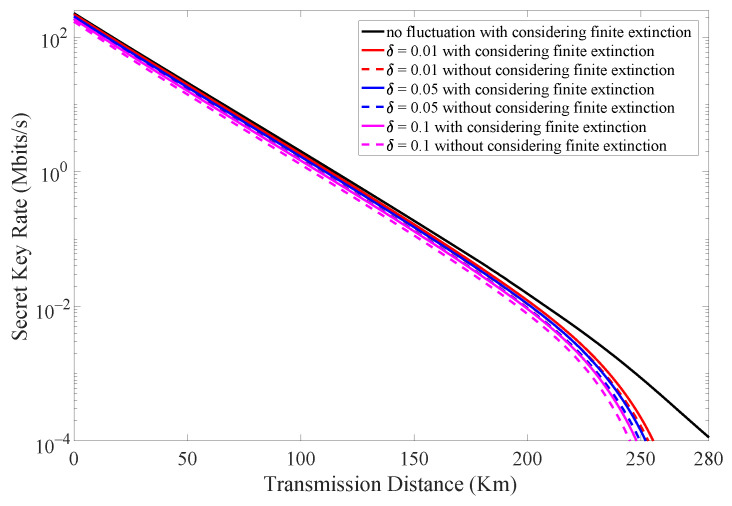
(Color online) The secret key rate vs. transmission distance when considering different intensity fluctuations (δ = 0.01, 0.05, 0.1) with and without considering finite extinction of intensity modulator.

The combined effect of finite extinction of intensity modulator and intensity fluctuation in laser source is illustrated in Figure 5. The number of transmitted signals is set to be $N=6.25\times 10^{11}$. We find that there is always an increase in the secret key rate when taking the finite extinction into consideration. Moreover, this improvement is more obvious when the intensity fluctuation increases. Table 3 shows different secret key rate results at a fixed transmission distance when considering different intensity fluctuations with and without considering the finite extinction of the intensity modulator.

## 5. Conclusions

In summary, we analyze the influence of the realistic intensity modulator on the practical security of high-dimensional quantum key distribution systems. We present finite-key analysis of HD-QKD with extinction ratios and intensity fluctuations. In our analysis, we improved the lower bounds of the secret key rate for the HD-QKD system with both the single photon state and the decoy-state method. We should also mention that different extinction ratios and intensity fluctuations have deep influences on the practical security of the HD-QKD protocol, and these issues are worthy of deep consideration when building realistic HD-QKD systems.

Furthermore, we should note that we conduct our analysis only in the time-bin HD-QKD system, and our method can be extended to HD-QKD systems by employing other different photonic degrees of freedom. Last but not least, our research has opened up a new path for the security analysis of practical HD-QKD systems. Analysis on other practical issues can follow the routine we proposed in this paper.

## Figures and Tables

**Table 1:** The maximal tolerable QBER with and without considering the finite extinction of intensity modulator for different dimensions.

Dimension	Maximal Tolerable QBER	
	without Considering the Finite Extinction of IM	with Considering the Finite Extinction of IM
d = 4	18.93%	19.27%
d = 8	24.71%	25.47%
d = 16	28.97%	30.58%

**Table 2:** The secret key rate calculated with and without considering the finite extinction of intensity modulator in units of Mbps.

Transmission Distance (km)	without Considering the Finite Extinction of IM	with Considering the Finite Extinction of IM
30	49.54	54.09
80	4.703	5.171
130	0.4417	0.4869
180	0.03927	0.04348
230	0.0027	0.00297

**Table 3:** The secret key rate results calculated with and without considering the finite extinction of intensity modulator for different intensity fluctuations when the transmission distance is 50 km in units of Mbps.

Intensity Fluctuation	0.01	0.05	0.1
Secret key rate without considering the finite extinction of IM	17.87	16.22	14.13
Secret key rate with considering the finite extinction of IM	19.67	18.03	15.96
Improvement	10.07%	11.16%	12.96%

## Data Availability

Not applicable.
